# Supplementary material for: MicroRNA-210 Suppresses NF-κB Signaling in Lipopolysaccharide-Stimulated Dental Pulp Cells Under Hypoxic Conditions
Source: Int J Mol Sci. 2025 Nov 7;26(22):10837. doi: 10.3390/ijms262210837 (PMC12652713; doi:10.3390/ijms262210837)
Supplement: Supplementary file 1 [file ijms-26-10837-s001.zip › ijms-3949371-supplementary.pdf]

Table S1. GeneChip miRNA 4.0 Array Data

Transcript ID (Array Design)	Ratio	P-value
**hsa-miR-210-3p**	**23.925**	**0.0003**
hsa-miR-1281	4.097	0.0480
hsa-miR-378a-3p	3.460	0.0003
hsa-miR-629-5p	3.091	0.0074
hsa-miR-532-5p	2.447	0.0005
hsa-miR-6867-5p	2.156	0.0048
hsa-miR-629-3p	2.130	0.0369
hsa-miR-18a-5p	2.088	0.0258
hsa-miR-216a-5p	1.993	0.0429
hsa-miR-202-3p	1.970	0.0488
hsa-miR-106a-5p	1.891	0.0494
hsa-miR-6850-5p	1.842	0.0344
hsa-miR-3185	1.790	0.0431
hsa-miR-5699-5p	1.735	0.0152
hsa-miR-19b-3p	1.696	0.0483
hsa-miR-509-3-5p	1.680	0.0137
hsa-miR-210-5p	1.658	0.0155
hsa-miR-4524b-5p	1.655	0.0197
hsa-miR-6089	1.648	0.0211
hsa-mir-3714	1.644	0.0005
hsa-mir-6891	1.638	0.0004
hsa-miR-106b-5p	1.612	0.0171
hsa-miR-542-3p	1.607	0.0276
hsa-miR-6090	1.576	0.0346
hsa-miR-4530	1.576	0.0128
hsa-miR-4454	1.575	0.0328
hsa-miR-6088	1.566	0.0140
hsa-miR-3663-3p	1.559	0.0467
hsa-miR-4516	1.549	0.0373
hsa-mir-514b	1.514	0.0383
hsa-miR-31-5p	1.511	0.0434
hsa-mir-1270-1	1.503	0.0384
hsa-mir-1270-2	1.503	0.0384

miRNA expression profile of the hDPCs (2×10^5 cells) cultured under normoxic (control, n = 3) or hypoxic (n = 3) conditions was examined using the GeneChip miRNA 4.0 Array (Thermo Fisher Scientific), and the miRNAs whose gene expression was upregulated by 1.5-fold or more under hypoxic conditions are listed.
